# Synthesis and Characterization of Polyhydroxyalkanoate/Graphene Oxide/Nanoclay Bionanocomposites: Experimental Results and Theoretical Predictions via Machine Learning Models

**DOI:** 10.3390/biom13081192

**Published:** 2023-07-30

**Authors:** Elizabeth Champa-Bujaico, Ana M. Díez-Pascual, Pilar García-Díaz

**Affiliations:** 1 Universidad de Alcalá, Departamento de Teoría de la Señal y Comunicaciones, Ctra. Madrid-Barcelona Km. 33.6, 28805 Alcalá de Henares, Madrid, Spain; elizabeth.champa@uah.es (E.C.-B.); pilar.garcia@uah.es (P.G.-D.); 2 Universidad de Alcalá, Facultad de Ciencias, Departamento de Química Analítica, Química Física e Ingeniería Química, Ctra. Madrid-Barcelona Km. 33.6, 28805 Alcalá de Henares, Madrid, Spain

**Keywords:** hybrid nanocomposites, graphene oxide, nanoclay, green synthesis, mechanical properties, machine learning, biomedical applications

## Abstract

Predicting the mechanical properties of multiscale nanocomposites requires simulations that are costly from a practical viewpoint and time consuming. The use of algorithms for property prediction can reduce the extensive experimental work, saving time and costs. To assess this, ternary poly(hydroxybutyrate-co-hydroxyvalerate) (PHBV)-based bionanocomposites reinforced with graphene oxide (GO) and montmorillonite nanoclay were prepared herein via an environmentally friendly electrochemical process followed by solution casting. The aim was to evaluate the effectiveness of different Machine Learning (ML) models, namely Artificial Neural Network (ANN), Decision Tree (DT), and Support Vector Machine (SVM), in predicting their mechanical properties. The algorithms’ input data were the Young’s modulus, tensile strength, and elongation at break for various concentrations of the nanofillers (GO and nanoclay). The correlation coefficient (*R*^2^), mean absolute error (*MAE*), and mean square error (*MSE*) were used as statistical indicators to assess the performance of the models. The results demonstrated that ANN and SVM are useful for estimating the Young’s modulus and elongation at break, with *MSE* values in the range of 0.64–1.0% and 0.14–0.28%, respectively. On the other hand, DT was more suitable for predicting the tensile strength, with the indicated error in the range of 0.02–9.11%. This study paves the way for the application of ML models as confident tools for predicting the mechanical properties of polymeric nanocomposites reinforced with different types of nanofiller, with a view to using them in practical applications such as biomedicine.

## 1. Introduction

Biopolymers are a foremost class of functional materials suitable for many applications, and they are currently of great interest to researchers and experts in numerous disciplines, particularly in the biomedical field. Amongst the widespread variety of biopolymers, polyhydroxyalkanoates (PHAs) have gained singular interest owing to their natural origin [1]. They are a family of fully biodegradable and biocompatible polyesters synthesized through bacterial fermentation from renewable resources. The simplest and most investigated PAH is poly(3-hydroxybutyrate) (PHB) [2], which possesses properties comparable to those of conventional synthetic plastics like polypropylene (PP). Nonetheless, PHB suffers from a number of shortcomings, including low impact resistance, poor thermal stability, and high water vapor permeability. To address these issues, it can be copolymerized with other PAHs, like polyhydroxyvalerate, leading to a copolymer named poly (hydroxybutyrate-co-hydroxyvalerate) (PHBV) with a chemical formula of [COCH_2_CH(CH_3_)O]_m_[COCH_2_CH(C_2_H_5_)O]_n_.

Graphene oxide (GO), a 2D nanomaterial obtained via oxidation of graphene, presents numerous surface oxygen groups, including carboxylic acids on the borders of the basal planes and hydroxyl and epoxy groups on the basal planes [3]. It presents outstanding thermal and mechanical properties, very large surface area, high optical transparency, and good biocompatibility, making it suitable for fields like medicine [4], high-performance composites [5], and chemical sensors [6]. However, conventional approaches to synthesizing GO, like Hummers’ method, involve time-consuming procedures and require aggressive reagents and toxic organic solvents, as well as carefully controlled operating temperatures, which lead to expensive GO when it is synthesized at an industrial level. In this regard, in a former study [7], we described a simple environmentally friendly and cheap two-stage approach for the synthesis of GO using an electrochemical cell that allowed the level of GO oxidation and exfoliation to be finely controlled by carefully modifying the synthesis conditions. 

On the other hand, nanoclays are nanoparticles of layered mineral silicates that possess outstanding mechanical properties. They provide composites with improved properties owed to their stability, swelling capacity, interlayer spacing, elevated hydration, and high chemical reactivity. One of the most used nanoclays is montmorillonite [8], with a chemical formula of [(Na,Ca)_0.33_(Al,Mg)_2_(Si_4_O_10_)(OH)_2_·nH_2_O], which consists of stacked layers with each layer composed of two O-Si-O tetrahedral sheets sandwiching one O-Al(Mg)-O octahedral sheet (ca. 100 nm × 100 nm, in width and length). This nanoclay has superior overall properties compared to traditional additives and fillers, for example, strength, stiffness, optical clarity, and permeability [9], and has been successfully used as reinforcement in polymeric matrices. 

To further improve the properties of polymeric materials, different nanofillers can be incorporated. Hybridization involving the combination of two nanofillers in a polymer matrix results in the reduction of water absorption and improved mechanical properties due to synergistic effects [10]. For instance, graphene/nanoclay hybrids have already been used as reinforcement in polymeric matrices [11]. It was found that in the absence of clay, aggregation of graphene nanoplatelets took place, while in the presence of clay, the nanoplatelets were better dispersed, resulting in improved electrical conductivity and mechanical properties. The effect of organic nanoclay Closite 20A and GO on the mechanical properties of polycarbonate nanocomposites has also been investigated [12] The results showed that the presence of nanoparticles aided in the dispersal of GO and improved the elastic modulus, flexural modulus, and compressive and tensile strengths. Even nanocomposites with three nanofillers, such as GO, nanoclay, and carbon nanotubes (CNTs), have been developed [13]. The hybridization between the three components synergized the mechanisms of mechanical resistance, with the optimum loadings of 3 wt% nanoclay, 0.05 wt% CNT, and 0.5 wt% GO. In the present study, ternary nanocomposites based on a PHBV matrix incorporating different amounts of GO and montmorillonite nanoclay were prepared via an easy, inexpensive, and sustainable solvent casting method to enhance the mechanical properties of PHBV matrix. By tuning the percentages of both nanofillers, the stiffness and strength could be carefully tailored to attain specific mechanical properties for targeted applications.

Machine learning (ML) is a subarea of artificial intelligence (AI). AI is a field of computer science that involves a set of technologies or tools that imitate cognitive functions associated with the human mind [14]. Its starting point is considered to be 1950, when the British mathematician and computer scientist Alan Turing published a paper entitled “Computing Machinery and Intelligence” in which he proposed the “Turing Test”, a test that measured the ability of a machine to exhibit intelligent behavior equivalent to or indistinguishable from human behavior [15].

Over the last 10 years, AI has become a tool increasingly used in many fields of science [16]. It allows researchers to analyze large amounts of data and complex models with high accuracy and efficiency, leading to important advances in different scientific fields, such as drug discovery [17] and molecular biology [18]. In AI, predictive models are systems based on machine learning algorithms that allow the model to learn from the data provided and adjust its parameters to achieve predictions that are more accurate. This capability makes AI a valuable tool for modeling the mechanical properties of nanomaterials, which is fundamental for their design and optimization. AI can analyze large experimental datasets and predict the properties of nanocomposites based on their composition and structure, leading to optimization of the design and manufacture of materials with specific properties.

There are three main types of AI: (1) Weak AI, referring to systems designed to perform specific and limited tasks. The goal is to find an automated solution to a problem or inconvenience or simply to improve something that already works but could work better. (2) General AI, which aims to teach the machine to understand and reason on a broader level, without being limited by its original programming, just as a human being would do. (3) Strong AI, in which the machine is intended to be functionally equal to a human being [19]. Currently, we are in an era of weak AI, as most AI systems are focused on a specific task. Although general and strong AI remain long-term goals, advances in AI technology are becoming significant and there are steady improvements in the efficiency and accuracy of AI systems. It is expected that, in the coming years and decades, AI will continue to improve and be applied to a wider range of problems and tasks [20].

**Scheme 1:**
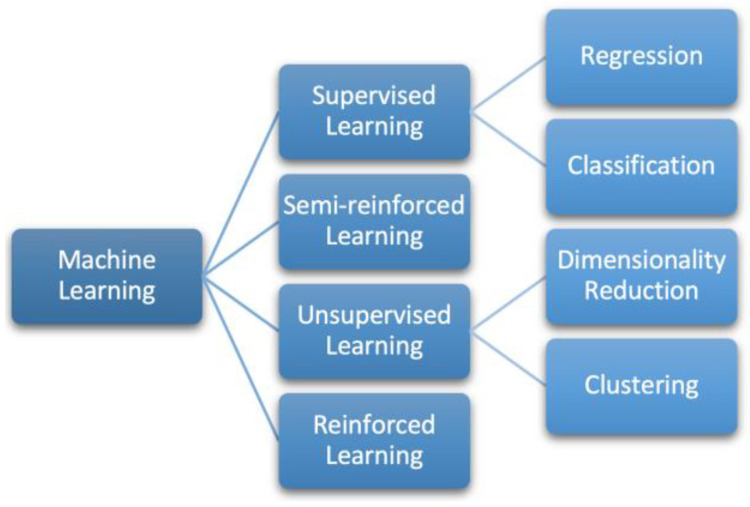
Types of ML. A general overview of different ML approaches.

ML, as a part of AI, has been widely applied in different fields, particularly in materials science. It uses mathematical algorithms to create predictive models. These algorithms analyze datasets, learn from the recurring patterns found, and make predictions or decisions based on that data [21]. Four main types of ML have been proposed (Scheme 1) according to the way in which the learning is performed and the type of data used: (1) Supervised learning, which generates models to predict output data from labeled data, i.e., based on historical examples of that output variable [20]. (2) Unsupervised learning, which has no labeled data, so the model clusters the data based on the hidden features underlying the data. (3) Reinforced learning, which is based on the principles of behavioral psychology, and the machine guides its own learning through rewards and sanctions in order to achieve the best possible result. (4) Semi-supervised learning, in which the model is trained using both labeled and unlabeled data [22].

Despite traditional equation-based approaches have been successfully used to predict the mechanical properties of polymer composites, it is important to mention that the approximations and results obtained using traditional methods may be less accurate and effective than those obtained using predictive ML models. The rule of mixtures is a commonly used equation for estimating the properties of a composite material, such as stiffness or strength, by assuming that they are a linear combination of those of the individual components [23]. However, this equation does not take into account non-linear and complex effects that influence the final mechanical properties, such as the aspect ratio of the filler, its orientation, and packing density. To address some of the limitations of the mixing rule, Krenchel mixing, which considers filler length, orientation, and distribution of the reinforcement particles in the composite, has been proposed [24]. However, Krenchel mixing can also have limitations and may not consider all of the factors that influence mechanical properties (i.e., polymer–filler interface, particle size, and surface area). Therefore, it is necessary to consider more advanced approaches, such as predictive ML models, which can capture non-linear and complex relationships in the data to obtain more accurate and effective results in estimating the mechanical properties of composite materials.

Recently, several studies have utilized ML models to predict the mechanical properties of polymeric nanocomposites [25]. Khanam et al. [26] predicted the tensile strength of linear low-density polyethylene (LLDPE)/graphene nanoplatelets (1–10 wt%) nanocomposites processed by twin extrusion via an artificial neural network (ANN). The average relative error in predicting the tensile strength of the nanocomposites with the ANN model was found to be very low (0.0396), demonstrating that the predicted results matched the experimental data. Using the same model, Zakaulla et al. [27] predicted the hardness, tensile strength, modulus of elasticity, and tensile elongation of polyetheretherketone (PEEK) nanocomposites containing graphene (2–10 wt%) and titanium powder (1–5 wt%) manufactured by injection molding, and the correlation factor between the training and testing datasets was higher than 0.9. Similarly, Kosicka et al. [28] predicted the same mechanical properties of epoxy/alumina (5–25 wt%) nanocomposites, and the model was found to be very effective (63% of predictions were very accurate, 15% were accurate, 20% were acceptable, and only 2% were unacceptable). Amani et al. [29] applied a linear ML-based regression model to predict the temperature-dependent Young’s modulus of epoxy/graphene nanocomposites. However, the application of ML in the study of polymer-reinforced nanocomposites is still in its infancy. Furthermore, the “black box” nature of ML usually does not provide the explicit relationships between inputs and outputs, which makes it difficult to apply, particularly in engineering design processes. Further, two frequent issues are encountered when applying ML to nanocomposite materials: overfitting and underfitting. The former occurs when the model notices the noise or random fluctuations in the training data and takes them as ideas, which weakens the capacity of the model for generalization. The opposite is underfitting, in which the model loses accuracy if it is trained with insufficient quantities of data. 

Hybrid nanomaterials are designed by combining two or more reinforcements at the nanometer scale and embedding them in a macroscale polymer matrix. These multiscale nanocomposites typically gain properties that can be tuned by the unique physical and chemical properties of the individual parts. Prediction of the mechanical properties of this type of hybrid is crucial for more efficient design of future structures with a view to using them in practical applications such as biomedicine. In this work, novel PHBV/GO/nanoclay bionanocomposites were prepared via a simple and environmentally friendly electrochemical process followed by solution casting using glacial acetic acid as an alternative green solvent, and their mechanical properties were investigated. Further, three ML-based algorithms were applied to predict their mechanical performance. The basis of each model is explained in detail in the next section, and their minimum square error (*MSE*), mean absolute error (*MAE*), and coefficient of determination (*R*^2^) were compared to determine the optimum model for each of the studied properties. It was demonstrated that ML algorithms are appropriate for modeling mechanical parameters with a high degree of accuracy and are hence highly valuable tools for predicting the mechanical performance of polymeric nanocomposites. 

## 2. Methodology

The regression models used in this study were Artificial Neural Network (ANN), Decision Tree (DT), and Support Vector Machine (SVM). The main characteristics of each model are described below. These models are widely used in the scientific literature [30,31]. We have selected them for their ability to model non-linear relationships between input and output variables, which is quite useful in predicting nanocomposite properties since they can depend on several factors. They also have good flexibility, adaptability, and predictive accuracy.

### 2.1. Artificial Neural Network 

**Scheme 2:**
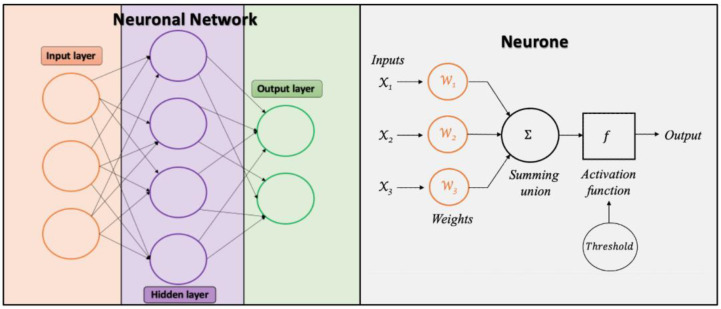
Structure of a neuronal network (**left**) and mathematical model of a neuron (**right**).

An ANN model is a type of ML algorithm based on the structure and functioning of the human brain [32]. These mathematical models can recognize patterns, learn from data, and make predictions based on these patterns. The basic structure of a neuronal network includes layers of interconnected neurons, each of which performs a mathematical operation on the input information and transmits it to the next layer. The first layer (input layer) gathers all the input data previously preprocessed, the intermediate or hidden layers carry out the calculations, and the last layer (output layer) offers the final predictions. A scheme of the basic structure of a neuronal network and a neuron is provided in Scheme 2. Similar to the brain, the network obtains knowledge from its environment through a learning process. Each neuron is connected to another and has an associated weight (inter-neuron connection strength or synaptic weight, which determines how information flows through the network) and a threshold (constant values that control the behavior of a neuron and improve the network’s ability to learn). 

During network training, weights and biases are adjusted so that the weights minimize prediction errors and biases in order to improve the network’s ability to model more complex relationships in the data [33]. At the output of each neuron in the network, activation functions are used to introduce non-linear relationships into the network. Both the numbers of layers and neurons directly influence the complexity of relationships in the data. The speed at which network parameters can be adjusted is controlled by the learning rate [34]. 

There are different ANN architectures [35]: (a) Feedforward neural networks are the simplest type of artificial neural network, in which information moves in only one direction, from input to output. These networks are composed of layers of neurons that process information sequentially [36]. (b) Recurrent neural networks, unlike feedforward networks, have connections between neurons that form loops, allowing information to flow in cycles within the network. This allows them to process sequences of data and possess short-term memory [37]. (c) Convolutional neural networks are networks designed to process matrix-structured data, such as images or audio signals. They use convolution operations to extract features from the data and reduce their dimensionality. They are typically composed of convolution and pooling layers that reduce the size of the data as the information moves through the network [38].

A feedforward network was used in this study because of its structure and ability to learn patterns, as well as its application in data regression. A neural network consists of an input layer, a hidden layer, and an output layer, and its choice was based on solid results obtained in the scientific literature [39,40].

In this study, the input data represented the chemical composition of the nanocomposites, and the output data depicted the corresponding mechanical properties. Each system was composed of two input variables related to the chemical composition of the nanocomposites (GO and montmorillonite nanoclay) and one output variable corresponding to a mechanical property (Young’s modulus, tensile strength, and elongation at break studied in independent networks). 

The Levenberg–Marquardt algorithm, commonly used in non-linear regression problems, was used to train the ANN. This method combines the gradient descent technique with the Gauss–Newton technique to minimize the error of the objective function. The architecture of the network can vary depending on the problem. In this case, it consisted of an input layer with two neurons, corresponding to the two input variables, a hidden layer that varied depending on the type of problem being tackled (which will be discussed in Section 4.3), and an output layer consisting of a single neuron for the output variable. The hidden layer used the activation function ReLU, a non-linear function very common in neural networks. As for the algorithm parameters, an initial learning rate of 0.09 and a maximum number of iterations of 1000 were defined. Although the learning rate may vary depending on the problem and the network architecture, a suitable value was chosen in this case. The maximum number of iterations was set to avoid overfitting by the model and to ensure that training was not unnecessarily prolonged. It is important to fit the model parameters appropriately, which may require a trial-and-error approach for each problem. 

The dataset was divided into training and testing data to adjust the weights and biases of the models and assess their accuracy and performance. Tensorflow functional API was used to implement the network, which allows more complex and flexible models for classification and regression tasks. The model was implemented in Python v.3.9.6 together with the Keras v.2.10.0 and Tensorflow v.2.10.0 libraries.

### 2.2. Decision Tree

DT is a supervised learning technique that operates in a tree-like model and is composed of three parts: the root node, the decision nodes, and the terminal or leaf nodes [41]. The model receives input data through the root node, and then the information flows through the branches of the tree, passing through the decision nodes according to the specific queries of each node, until it reaches the leaf nodes, which represent the final predictions. The tree construction process is performed by splitting, pruning, and processing the pruned tree to improve understanding, starting at the root node and progressing until a specific stop condition is met [42,43,44]. The splitting at each node is performed in such a way as to minimize the prediction error [45]. 

The tuning of hyperparameters that control the splitting and pruning of the tree can have a major impact on the accuracy and performance of the resulting model. For example, the maximum depth parameter controls the maximum depth of the tree and influences the complexity of the model and its ability to generalize to new data. Thus, if a large maximum depth is set, the model may overfit the data, while if a small maximum depth is set, the model may lead to poor generalization and low accuracy. Other important hyperparameters are: (a) the split criterion, which determines the strategy used to choose the split at each node; (b) minimum samples split, which considers the minimum number of samples required to split a node; (c) minimum samples leaf, which considers the number of samples required to be a leaf node; (d) maximum leaf nodes, which determines the maximum number of nodes a tree can have; and (e) maximum features, which represents the number of predictors considered in each split [46]. 

### 2.3. Support Vector Machine 

SVM is a supervised learning algorithm used in both classification and regression problems. In regression problems, SVM searches for a function that fits the input data, thereby minimizing the prediction error [47]. SVM handles non-linear data using kernel functions, which transform the input data into a higher dimensional space in which the data are more likely to be separable [48,49].

The main hyperparameters to be tuned are: (a) Regularization parameter C, which determines the trade-off between model complexity and the model’s ability to fit the training data. (b) the Kernel parameter, which determines the function used to transform the input data into a higher dimensional space, with the most common kernel types being the linear kernel, the polynomial kernel, and the radial basis function kernel. This study used the radial basis function kernel (RBF), a non-linear function commonly used in regression problems in which the data have a non-linear structure [50]. (c) Gamma, used in combination with the RBF kernel, which controls the shape of the kernel function and therefore has a significant impact on the accuracy and performance of the model. (d) The error tolerance parameter, epsilon, which controls the margin around the regression hyperplane [51].

Both the DT and SVM models used combinations of values of the different named hyperparameters, allowing the models to optimally learn from the data. Both were implemented using the Python V.3.9.6 programming language and the Scikit-learn v.0.23.2 library.

The mean absolute error (*MAE*), the mean square error (*MSE*), and the coefficient of determination (*R*^2^) were the statistical indicators used to assess the performance of the developed models. *MSE* was calculated as the mean of the squares of the differences between the predicted and actual values. It is defined as follows:

$$MSE=\frac{1}{n}\sum {\left(y^{\prime }-y\right)}^{2}$$

where *y*′ is the predicted value, *y* is the corresponding value, and *n* is the total number of samples in the dataset.

*MSE* penalizes large errors more than small errors due to the squaring operation, which can make the model more sensitive to outliers or grossly erroneous predictions.

*MAE* is calculated as the average of the absolute differences between the predicted and actual values:

$$MAE=\frac{1}{n}\sum \left|y^{\prime }-y\right|$$

*MAE* is less sensitive to outliers than *MSE* as it uses absolute values instead of squares. However, by not squaring the errors, *MAE* may underestimate the impact of large errors.

*R*^2^, the proportion of the variation in the dependent variable that is predictable from the independent variable(s), is calculated as:

$$R^{2}={\left(\frac{n(\sum yy^{\prime })-(\sum y)(\sum y^{\prime })}{\sqrt{\left[n\sum y^{2}-{\left(\sum y\right)}^{2}\right][n\sum {y^{\prime }}^{2}-{\left(\sum y^{\prime }\right)}^{2}]}}\right)}^{2}$$

*R*^2^ is a measure of goodness of fit that is commonly used to assess the accuracy of ML models. It represents the degree of correlation between the model prediction and the target values. The accuracy of a model improves as *R*^2^ approaches 1. 

## 3. Experimental

### 3.1. Material

Poly(3-hydroxybutyrate-co-3-hydroxyvalerate) (PHBV) with M_n_ = 84,000 g/mol, T_g_ ≈ 23 °C, T_m_ ≈ 165 °C, d_25 °C_ = 1.25 g/cm^3^, and HV content of 12 mol% was purchased from Goodfellow Corp. Flexible graphite foil (FGF) with d_25 °C_ = 1.00 g/cm^3^, C: 99.5%, S < 300 ppm, Cl < 50 ppm, ash < 1%, and thickness 0.1 mm was supplied by Beyond Materials, Inc. (Tucson, AZ, USA) and dried under vacuum for 48 h before use. Glacial acetic acid (C_2_H_4_O_2_, >99%) and k1_0_ montmorillonite powder (97%, 220–270 m^2^/g) were provided by Sigma-Aldrich. Ultrapure water was purified using a Millipore system (Millipore, Burlington, MA, USA).

### 3.2. Nanocomposite Preparation

First, GO was synthesized from FGF via a two-step electrochemical process at room temperature [7]. The process was accomplished in an electrolytic cell with a slice of FGF fixed onto a tungsten wire with silver glue as the anode, a Pt wire as the cathode, and 98 wt% H_2_SO_4_ diluted in 100 mL of Milli-Q water as the electrolyte. Initially, a voltage of 2 V was applied for 10 min, leading to the formation of a graphite intercalation compound (GIC). Then, the GIC was oxidized via application of high voltage (20 V) for 1 min. The synthesized GO was collected by filtration, washed with water, purified via centrifugation at 2500 rpm, and ultrasonicated for 30 min at a power of 140 W. The resulting GO had a C/O ratio of 1.46 according to elemental analysis [7].

The nanocomposites were prepared via solution casting, following a multi-step process. First, the required amount of GO was dispersed in deionized water via bath sonication for 20 min. Separately, the required amount of montmorillonite powder was dispersed in water by sonication for 10 min and then added to the GO dispersion, which was sonicated for 60 min to attain an exfoliated mixture. Separately, the PHBV powder was dissolved in glacial acetic acid at 60 °C, added to the GO/nanoclay dispersion, and then the mixture was sonicated for another 60 min at room temperature. The ternary mixture was then cast onto a glass Petri dish and finally dried for 2 days under vacuum. 

**Scheme 3:**
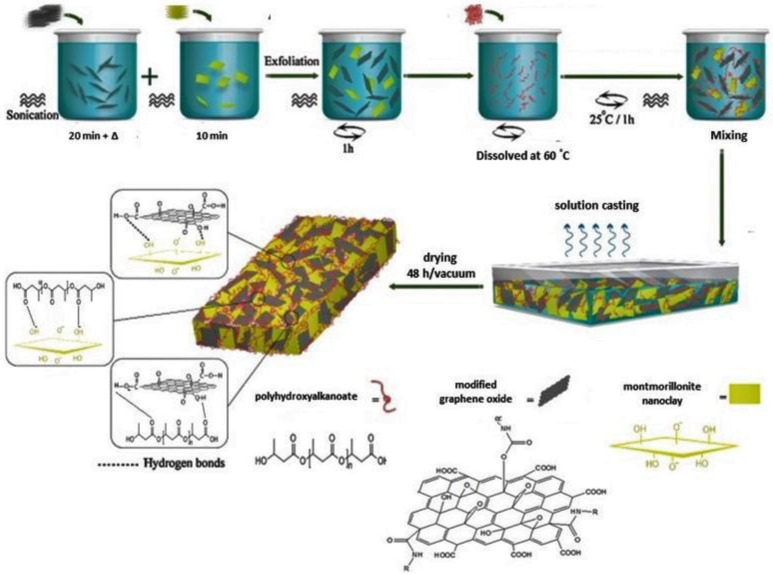
Representation of the synthesis procedure of the nanocomposites and potential interactions between components.

A schematic representation of the synthesis of the nanocomposites, the chemical formula of each component, and the interactions between them via hydrogen bonding is shown in Scheme 3.

### 3.3. Characterization Techniques

All of the samples were conditioned for 24 h before the measurements. The tensile strength tests were carried out following the ASTM D 638-03 standard on a servo-hydraulic testing machine (858 Mini Bionix; MTS Systems Corporation, MN, USA) at a crosshead speed of 1 mm/min, with a load cell of 100 kN, at 23 °C, and under 50% RH. 

Charpy impact strength tests were carried out according to the ASTM D 6110-10 standard under the same environmental conditions on a CEAST Fractovis dart impact tester (Instron, MA, USA). Notched specimen bars and a hammer mass with an energy of 7.10 J were used. Five specimens for each type of nanocomposite were measured to check for repeatability, and the average value is reported. 

The surface morphology of the nanocomposites was examined by scanning electron microscopy (SEM) using a scanning electron microscope (SIGMA VP-500; Zeiss, Germany) at an acceleration voltage of 10 kV. Samples were first cryo-fractured and then sputtered with a gold layer under vacuum to avoid charging during electron irradiation.

## 4. Results and Discussion

### 4.1. Surface Morphology of PHBV/GO/Nanoclay Composites

**Figure 1:**
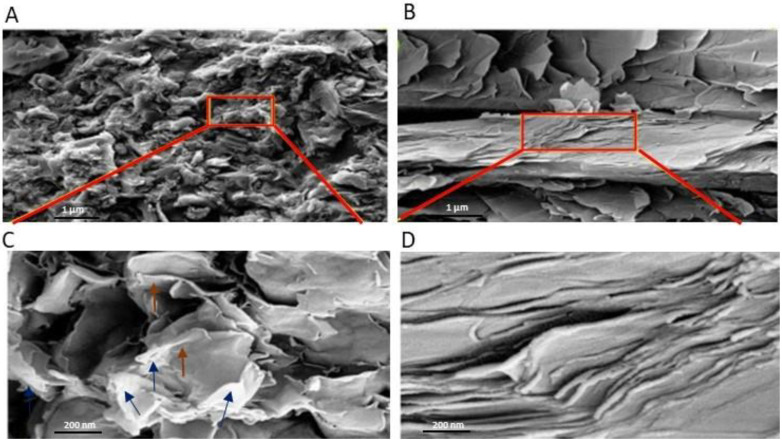
SEM images from fractured surfaces of PHBV/GO (2 wt%)/nanoclay (2 wt%) nanocomposites at different magnifications. Scale bar 1 µm (**A**,**B**). Scale bar 200 nm (**C**,**D**). The red brackets in (**A**,**B**) indicate the zoomed region. The blue and brown arrows in (**C**) point out graphene oxide and nanoclay sheets, respectively.

The morphology of the hybrid PHBV-based nanocomposites was investigated using SEM, and typical images at different magnifications of the cross-section of the sample with 2 wt% GO and 2 wt% nanoclay are shown in Figure 1. Similar morphologies were observed for the other hybrid composites. The nanocomposites showed a 3D structure with a multilamellar morphology consisting of GO flakes and montmorillonite nanosheets embedded into the continuous semicrystalline phase of PHBV. Intercalated sheets of both nanomaterials could be observed; the most rigid sheets corresponded to the nanoclay whilst the most flexible and fluffy sheets belonged to the modified graphene oxide. In the images at lower magnification (Figure 1A,B), both nanofillers were found to be homogenously and randomly dispersed into the matrix, forming a dense and entangled network, likely due to hydrogen bonding interactions between the three nanocomposite components (see Scheme 3). Further, other interactions, such as polar and hydrophobic interactions, may have contributed to the formation of a reinforced network that strongly adhered to the matrix. In the images at higher magnification (Figure 1C,D), nanoclay nanosheets about 20 nm thick were found to be surrounded by exfoliated and wrinkled graphene nanosheets with an average thickness of 6 nm. 

### 4.2. Experimental Mechanical Properties of PHBV/GO/Nanoclay Composites

**Figure 2:**
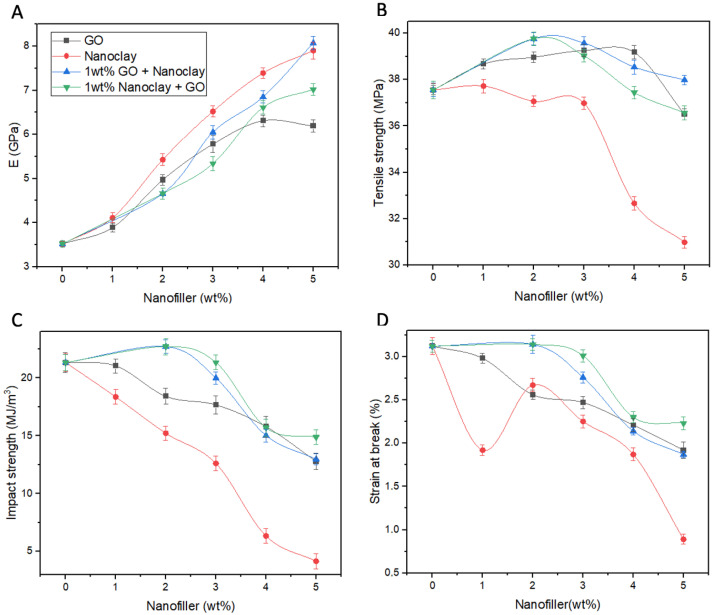
Mechanical properties of PHBV/GO/nanoclay nanocomposites as a function of total nanofiller loading: Young’s modulus (**A**), Tensile strength (**B**), impact strength (**C**) and elongation at break (**D**). The black and red lines correspond to binary nanocomposites reinforced with GO and nanoclay, respectively. The blue and green lines correspond to ternary nanocomposites reinforced with 1 wt% GO or 1 wt% nanoclay, respectively, and different amounts of the other nanofiller.

The results of the tensile and Charpy notched impact strength tests are presented in Table 1, and the data as a function of total nanofiller loading are plotted in Figure 2.

The neat copolymer had a Young’s modulus (E) value of ∼3.5 GPa, which rose gradually with increasing nanofiller loading up to 76% and 125% after the addition of 5 wt% GO or nanoclay, respectively (Figure 2A). Regarding the binary nanocomposites with nanoclay, the rise was almost linear within the concentration range studied, suggesting that these nanofillers were well dispersed within the matrix, and hence had a very effective reinforcement effect. The increments found herein were significantly higher than those reported previously for PHBV nanocomposites filled with organo-modified montmorillonite prepared via melt blending, probably due to the partial degradation of the polymer during processing [52]. They were also much higher than those found upon addition of spherical inorganic nanofillers to PHBV [53], which was attributed to the fact that spherical nanoparticles enable more free volume space between them, allowing the polymer segments to deform in a more mobile manner [11]. In contrast, the modulus of the binary nanocomposites with GO decreased slight for the highest concentration, likely due to a slight aggregation of the GO flakes at higher loading, which reduced their effective surface area and hence interaction with the matrix. This issue was solved in the ternary nanocomposites, which showed a linear rise with increasing nanofiller loading, particularly those with 1 wt% GO and increasing amounts of nanoclay. Thus, the nanocomposite with 1 wt% GO and 4 wt% nanoclay showed the highest modulus amongst all of the nanocomposites, indicating a synergistic effect of both fillers to enhance the mechanical properties of the matrix. These results were consistent with those previously reported for hybrid nanocomposites reinforced with nanoclay and GO, such as those based on a polycarbonate matrix [12] in which the optimal weight fractions for the two nanofillers were 1% and 0.6%, respectively. Higher fractions led to the formation of agglomerates and degradation of the nanocomposites. Similarly, the effect of graphene nanoplatelets and montmorillonite nanoclay on the mechanical properties of epoxy nanocomposites was previously explored [54] and optimal concentrations of 0.2 wt% of graphene and 3 wt% nanoclay were found, resulting in the highest Young’s modulus and strength improvements (about 19% and 18%, respectively). Notably, the increments found in this work were higher, likely because the strong interactions between the matrix and the nanofillers via hydrogen bonding, as mentioned earlier, resulted in the formation of a dense and entangled network (Figure 2) that in turn led to very effective reinforcement. Also, the solution casting method used herein that employed several cycles of ultrasonication enabled a very homogenous dispersion of the nanofillers; hence, the optimal concentrations of nanoclay and GO were higher than those in previous works, which was also reflected in stronger stiffness and strength enhancements. 

Very different behavior was observed for the tensile strength (Figure 2B). This property remained almost constant in the binary nanocomposites with nanoclay up to 3 wt% loading and then decreased, while it increased moderately with increasing GO loading until it reached a maximum value at 4 wt%, and then it decreased slightly. This behavior could be explained considering that in the nanocomposites with high nanoclay loading, the nanosheets confin the drawn fibration process of the matrix, leading to a strong reduction in ductility, as depicted in Figure 1D, and premature system failure, which was reflected in the lower tensile strength of these samples. It could also arise from strong hydrogen boding interactions between the polymer and the nanoclay due to the numerous hydroxyl groups on the montmorillonite surface. Further, this also resulted in a significant decrease in the impact strength, as shown in Figure 2C. Conversely, since GO has fewer hydroxyl groups, it imposes less restriction on polymer chain mobility, so the drops in ductility and impact strength were less pronounced. 

Regarding the ternary hybrids with 1 wt% GO and increasing amounts of nanoclay, tensile strength rose over the whole concentration range, and elongation at break and impact strength increased slightly at low nanofiller content and then were approximately maintained up to 3 wt% loading. In addition, those hybrids with 1 wt% nanoclay and increasing contents of GO preserved the ductility and toughness up to 3 wt% loading. This was very interesting since one of the main drawbacks of PHBV is its low impact strength. The nanoclay seemed to intercalate within the GO flakes, leading to a more exfoliated structure with a very large contact area with the matrix, hence inducing very strong interfacial adhesion, which was beneficial for improving or at least preserving polymer toughness. Overall, the hybrid nanocomposites with 1 wt% GO and increasing amounts of nanoclay showed the optimal balance of mechanical properties. 

The experimental mechanical properties of the PHBV/GO/nanoclay nanocomposites were first estimated using the traditional rule of mixtures [23], assuming that the volume fractions could be approximated by the weight fractions, since the density of GO was unknown. For example, taking literature stiffness values of 380 GPa for GO [55] and 178 GPa for nanoclay [56], the stiffness of the nanocomposite with 1 wt% GO and 1 wt% nanoclay would be 9.03 GPa. However, the experimental value was 4.66 GPa, which showed a significant discrepancy. Similar behavior was found for the other ternary nanocomposites, with differences up to 95%. The Krenchel modified rule of mixtures for discontinuous reinforcement was also applied, considering a correction factor of 1/5 for randomly oriented nanofillers [24]. For the indicated nanocomposite, this method yielded a value of 4.556 GPa, which was very close to the experimental value. However, for the nanocomposite with 2 wt% GO and 3 wt% nanoclay, a value of 5.93 GPa was obtained, which differed strongly from the experimental value (8.76 GPa). Even larger differences between the experimental results and the theoretical predictions were found for the composites with higher loadings. 

The above examples illustrated that neither the rule of mixtures nor Krenchel mixing provided accurate approximations of the mechanical properties of the multiscale composites. This justifies the need for the development of more advanced approaches, such as predictive ML models, as will be discussed in the following sections. 

### 4.3. Predicted Mechanical Properties Using ML Regression Models

ANN, DT, and SVM were the models used in this research for the prediction of the mechanical properties of the nanocomposites as they have the ability to handle large datasets and non-linear data, generalize to new data, identify patterns, and improve prediction accuracy. To properly train these models, input data capturing the nanocomposite composition (GO and nanoclay weight percentages) and output data corresponding to the mechanical properties associated with these compositions (Young’s modulus, tensile strength and strain at the break) were required. It is important to mention that the input data were not normalized herein, which meant that no transformation was performed to adjust the scales of the features or input variables. In addition, cross-validation with the training set was performed using a k-fold cross-validation approach with k equal to 5. For the data split, 75% was used for model training and 25% was used for testing and evaluation. This configuration allowed us to evaluate the performance of the model based on unseen data and to estimate their generalizability. It is important to find the best fit of the hyperparameters in the model training stage. These are fixed values selected before the model run that affect the performance of the model. In the case of the ANN model, the number of neurons in the hidden layer was adjusted to optimize the performance of the network. In the DT model, the hyperparameters to be adjusted included the maximum depth of the tree, the minimum number of samples required to split an internal node (minimum samples split), the minimum number of samples required for a terminal node (minimum samples leaf), the maximum number of terminal nodes (maximum leaf nodes), and the maximum number of features for each split and the criterion used to split the nodes (splitter). Finally, for the SVM model, the values of the regularization parameter C and the kernel, gamma, and epsilon parameters had to be adjusted.

Before the final training of the predictive models, a manual tuning process of the hyperparameters was carried out. During this process, some of the values were selected using previous knowledge and experience, while others were based on previous studies that had obtained encouraging results using the same values. The combination of manual adjustment and reference to previous studies was crucial in establishing the final configuration of each predictive model [57,58,59,60].

Furthermore, the search for the optimal hyperparameter combination was supported by the evaluation metrics of the predictive models, in which precision is one of the most used metrics. In the following sections, the coefficient of determination obtained from various configurations of hyperparameters in different models are presented.

#### 4.3.1. Young’s Modulus

**Figure 3:**
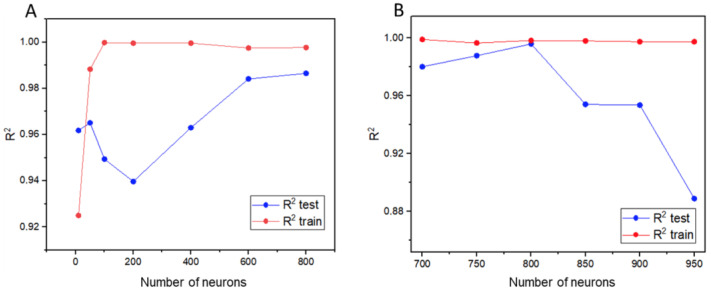
Relationship between the coefficient of determination (*R*^2^) and the number of neurons in the hidden layer of the ANN model for the prediction of Young’s modulus for the training and testing sets. (**A)** Range: 0–800 neurons; (**B**) Range: 700–950 neurons.

The effect of the number of neurons in the hidden layer on the performance of the ANN was evaluated. Figure 3 shows the change in the coefficient of determination (*R*^2^) of the training and testing sets as a function of the number of neurons in the hidden layer. 

Figure 3A shows that in the training data, the value of *R*^2^ increased up to 100 neurons and then remained almost constant, while in the testing set, it decreased up to 200 neurons and then rose. Increasing the number of neurons in the hidden layer can improve the fit of the model to the training data, as the model increases the ability to learn complex patterns in the data. Thus, above 600 neurons, the study region was delimited, and the best accuracies in the training and testing sets were observed for 800 neurons. However, this increase can also increase the risk of overfitting by the model and decrease its ability to generalize to new data, as occurred with neuron counts above 800 (Figure 3B). Above 800 neurons, a decrease in the accuracy of the testing set was also observed, reflecting an overfit of the training data, which resulted in a model with poor performance that did not generalize well to new data. A trade-off between model fit and generalization was found at 800 neurons, with high training and testing set accuracy values of 0.9984 and 0.9960, respectively. These values were higher than those obtained in the study by Ho et al. [61], who proposed an ANN model for the prediction of Young’s modulus of polymer/carbon nanotube composites and obtained training and testing set coefficient of determination values of 0.986 and 0.978, respectively. The results were also better than those reported for LLDPE/graphene nanoplatelets composites, in which the best results for Young’s modulus showed an *R*^2^ value of 0.933 [62], and those found for ternary PEEK/Ti/C nanocomposites, with an *R*^2^ value of the training set equal to 0.96 [26].

**Figure 4:**
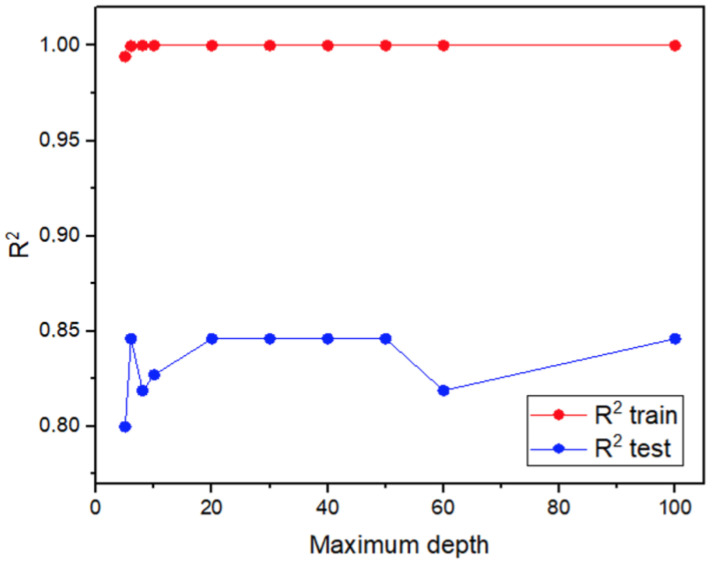
Relationship between *R*^2^ and the depth of the DT model for the prediction of Young’s modulus for the training and testing sets.

The tree depth adjustment in the DT model is analyzed in Figure 4, which shows the variation in the coefficient of determination as a function of the tree depth hyperparameter. A trend was observed for both the training and testing sets around 20 and 50 tree depths, which indicated that the model had reached its learning capacity limit, and it was not possible to improve its performance by adjusting only this hyperparameter. Increasing the tree depth meant allowing more splits of the data, capturing complex patterns, reducing the error of predictions, and more closely matching the training data. The maximum accuracy values obtained in this first assessment were 1.0 and 0.8461 for the training and testing sets, respectively. From a value of 50 onwards, there was a loss of generalization to the training data. 

Table 2 shows that increasing values for the minimum number of samples to split an internal node and for a leaf node reduced the ability of the model to fit the data, which also occurred with low values for the maximum number of nodes in a leaf. An increase in the minimum number of samples required to split an internal node results in fewer splits in the tree (or an increase in the minimum number of samples required for a terminal node), as increasing the number of samples results in a model that is insufficiently flexible to capture small variations in the data. In addition, decreasing the maximum number of terminal nodes, i.e., a smaller size at the leaf node, may result in overfitting and increased sensitivity to noise. Therefore, it is important to find the right balance between the complexity of the model and its generalizability to new data. The “auto” option of the ‘Max. features’ parameter in Table 2 was the best choice for maximum accuracy, while the “best” or “random” options did not influence the accuracy. After evaluating different values for the hyperparameters, the best coefficient of determination values for the training and testing sets were 1.0 and 0.8461, respectively.

Regarding the SVM regression model, the hyperparameter C was evaluated since it is an important factor to balance the complexity of the model and its ability to generalize to new data [63]. Using high C values, the dataset was perfectly represented by our predictor hyperplane, although it yielded a more complex model. Conversely, low C values led to a simpler model, in which the margin violations were largely penalized. Therefore, it is important to find the right balance between model complexity and fitting accuracy [64]. 

**Figure 5:**
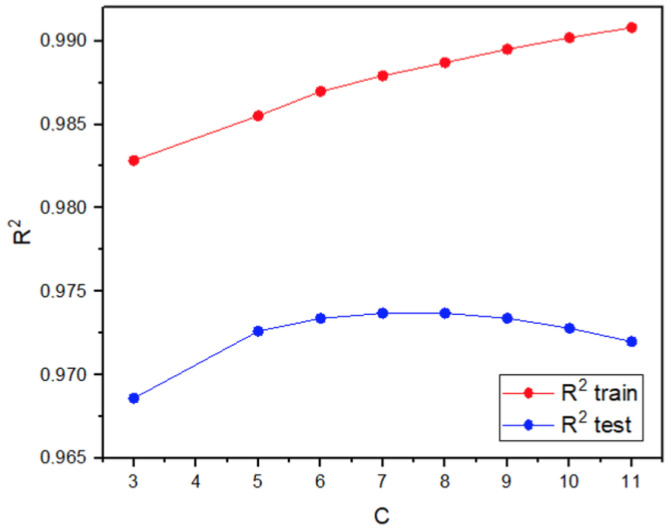
Relationship between *R*^2^ and the regularization parameter C in the SVM regression model for the prediction of the Young’s modulus.

Figure 5 shows the change in *R*^2^ of the training and testing sets as a function of the value of the C parameter. The coefficient of determination of the testing set rose with increasing C value until it reached a maximum value of 0.9728 for C equal to 7, and then it decreased. Conversely, *R*^2^ of the training set rose gradually with increasing C, showing a maximum value of 0.9902 for C equal to 11. This pattern suggest that 7 is the optimal C value that maximizes the performance of the model, and that SVM is therefore a reliable model for predicting the performance of the nanocomposites in terms of the two independent variables, the two nanofiller loadings [65]. This study achieved higher accuracy than that obtained with the SVM model (*R*^2^ of 0.835) reported by Wang et al. [66] for evaluating the performance of thin-film nanocomposite organic solvent nanofiltration membranes in terms of relative permeability and relative selectivity.

Zamanian et al. [51] optimized the mechanical properties of polyvinyl alcohol (PVA)/TiO_2_/montmorillonite nanocomposites using an SVM model. They found *R*^2^ values systematically higher than 0.96 for the testing data, indicating that SVM could be used as a reliable model for predicting nanocomposite performance in terms of two independent variables, the two nanofiller loadings. Similar conclusions can be drawn in our work, since the *R*^2^ values were higher than 0.97 for the testing data. 

#### 4.3.2. Tensile Strength

**Figure 6:**
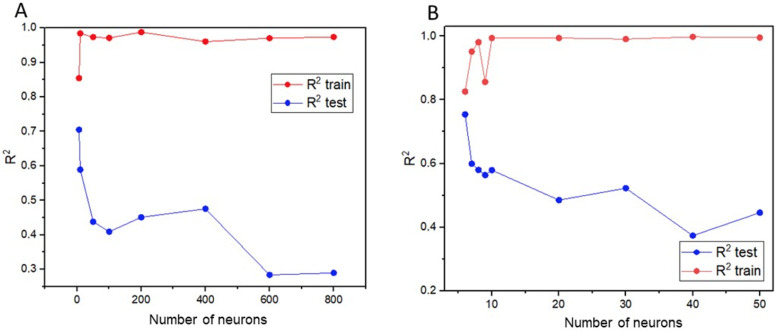
Relationship between *R*^2^ and the number of neurons in the hidden layer of a neural network for the prediction of the tensile strength for the training and testing sets. (**A**) Range: 0–800 neurons; (**B**) 5–50 neurons.

The first model assessed for the prediction of tensile strength was the ANN model. Figure 6 shows the coefficient of determination as a function of the number of neurons in the hidden layer of the ANN. The coefficient of determination of the training set remained almost constant with increasing number of neurons. Conversely, as the number of neurons increased, the loss of generalization to the testing data was higher (Figure 6A). The overfitting found can occur when a model performs well on the training set and captures random variability in the data rather than the underlying relationship between variables. The model may be too complex or have too many parameters for the size of the data. Therefore, a region with a smaller number of neurons was studied, as shown in Figure 6B. A maximum coefficient of determination (*R*^2^) value of 0.579 was found for 10 neurons in the hidden layer. Although this result exceeded the minimum value for an adequate model, it suggested that the model was not making good predictions on the testing data; thus, it was interesting to consider other predictive models such as DT or SVM.

The DT regression model was also applied to predict the tensile strength, and the *R*^2^ value as a function of the tree depth parameter is shown in Figure S1 in the Supporting Information. The *R*^2^ values of both the training and testing sets remained almost constant, indicating that the model could not improve its performance by only adjusting this hyperparameter. Thus, different values of the hyperparameters were tested (Table 3), and it was found that increasing the values for the minimum number of samples to split an internal node and the minimum number of samples required for a leaf node reduced the accuracy of the training and testing sets. On the other hand, increasing the value for the maximum number of nodes in a leaf increased this accuracy, reaching its maximum with a value equal to 100 for this parameter. In turn, the “auto” option for the maximum features parameter and the “random” option for the splitter parameter were the best alternatives to achieve better accuracy. The best values for the coefficient of determination were 1.0 and 0.9386 for the training and testing sets, respectively.

Regarding the SVM regression model, the plot of the coefficient of determination (*R*^2^) versus the C parameter showed an almost constant trend for both the training and testing sets (Figure S2 in the Supporting Information). A maximum *R*^2^ value of 0.73 in the testing set was obtained for a C parameter value equal to 140. However, this value was not enough for a suitable prediction model; hence, we proceeded to adjust the other hyperparameters of the model, epsilon and gamma, to improve its performance (Table 4).

The epsilon parameter controls the error between the data points and the regression line, so increasing its value allowed for a larger number of data points within the error band, causing a slight decrease in the value of the coefficient of determination of the training set. Simultaneously, it also produced an increase in the value of the coefficient of determination of the testing set by allowing better generalization of the model to new data up to a maximum value (*R*^2^ = 0.8293), and then the accuracy decreased. On the other hand, the gamma parameter had less influence on the improvement of the accuracy of the SVM model. It performed better for a default value of 0.1, showing a good balance between accuracy and generalization of the model. After adjusting both hyperparameters, the optimum values of *R*^2^ (0.9841 and 0.8293 for the training and testing sets, respectively) were obtained for an epsilon value equal to 0.35.

#### 4.3.3. Elongation at Break 

**Figure 7:**
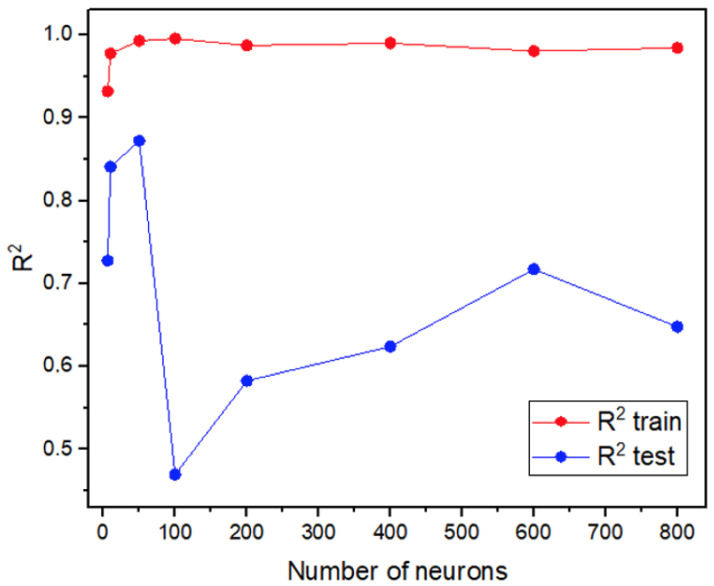
Relationship between *R*^2^ and the number of neurons in the hidden layer of a neural network for the prediction of elongation at break.

Figure 7 shows the change in *R*^2^ for the training and testing sets vs. the number of neurons in the hidden layer of the neural network. The *R*^2^ value of the training set remained almost constant, while the accuracy of the testing set generally increased, similar to the trend found for Young’s modulus, reaching a maximum accuracy with 50 neurons (*R*^2^ values of 0.9928 and 0.8723 for the training and testing sets, respectively). These results were better than those reported for ternary PEEK/Ti/C nanocomposites, with an *R*^2^ value of the training set equal to 0.94 [26].

The general trend found indicated that the model was generalizing well to new data, and that the training data were not being overfitted. 

Figure S3 in the Supporting Information shows the change in *R*^2^ for the training and testing sets vs. the value of the tree depth parameter. A constant trend was observed almost throughout the whole range of the parameter study. The single adjustment of this parameter was insufficient to improve the performance of this model; thus, the other hyperparameters were tuned and the results are included in Table 5. Increasing the minimum number of samples to split an internal node and the minimum number required for a leaf node reduced the accuracy, while increasing the number of leaf nodes improved the accuracy, reaching a maximum for a value of this parameter equal to 60 (*R*^2^ values of 1 and 0.8894 for the training and testing sets, respectively). For higher values, the accuracy decreased for two main reasons: overfitting and noise. On the other hand, the default “auto” option for maximum features and the “random” option for the splitter parameter maintained adequate accuracy. The latter is a more flexible option able to better adapt to the variability in the data, capturing more patterns in the data; however, it can also introduce noise and unnecessary variability in the model.

Regarding the SVM model, an almost constant *R*^2^ value was found with increasing C parameter value (Figure S4), particularly in the range of 20 to 100. However, the maximum *R*^2^ value obtained in the testing set was 0.780; hence, different hyperparameters such as epsilon and gamma were also evaluated to obtain an adequate prediction model (Table 6).

The *R*^2^ value of the testing set increased with increasing epsilon parameter value up to a maximum of 0.9127 for an epsilon value equal to 0.2, where the model achieved an appropriate balance between fit and generalizability. For higher values, a more restricted model was obtained, decreasing its ability to fit and, consequently, decreasing the value of the coefficient of determination. On the other hand, a gamma hyperparameter value equal to 0.1 was the most appropriate for the dataset in question, as the accuracy results possibly reflected that the model transformed the input data in a non-linear way, improving its capacity for effective dimensionality reduction. This simplified the model and improved the accuracy in predicting new data. The maximum values of the coefficient of determination for the training and testing sets were 0.9526 and 0.9127, respectively.

### 4.4. Error Assessment of Predictive Models. Measured vs. Predicted Values

To select the best model to predict each of the mechanical properties investigated, the coefficient of determination values were compared, since it is used as a measure of the ability of a model to explain the variability in the data. The *R*^2^ values for the three models applied are compared in Table 7. The higher the *R*^2^ value, the better the model, since it is able to explain a larger proportion of the variability in the data.

According to the data collected in Table 7, the best predictive model for the Young’s modulus was the ANN model. Similar conclusions were drawn from the study on graphene/aluminum nanocomposites carried out by Liu et al. [57], who compared the performances of ANN and SVM models in predicting the Young’s modulus of the nanocomposites. In this case, ANN with a hidden layer of 40 neurons showed the highest *R*^2^ and the minimum *MSE*. Regarding the tensile strength, the optimal model was the DT regression model, with *R*^2^ values of 1 and 0.9386 for the training and testing sets, respectively. However, for elongation at break, the best model was the SVM, with *R*^2^ values of 0.9526 and 0.9127 for the training and testing sets, respectively. This suggested that the optimal ML model depended on the specific mechanical property to be predicted and not on the nature of the system. Thus, the complexity of the nanocomposites studied, comprising three components, and the different factors affecting each mechanical property in addition to the different nanofiller loadings (i.e., matrix-filler adhesion, filler–filler interactions, matrix modulus, strength, ductility, filler modulus, etc.) made generalization difficult. Similar conclusions were drawn for the prediction of the mechanical properties of fiber-reinforced polymer composites using different ML algorithms [67]. Nonetheless, the differences in performance between the ANN and SVM models were relatively small, and both were successfully applied to predict the Young’s modulus of the hybrid nanocomposites developed herein. 

**Figure 8:**
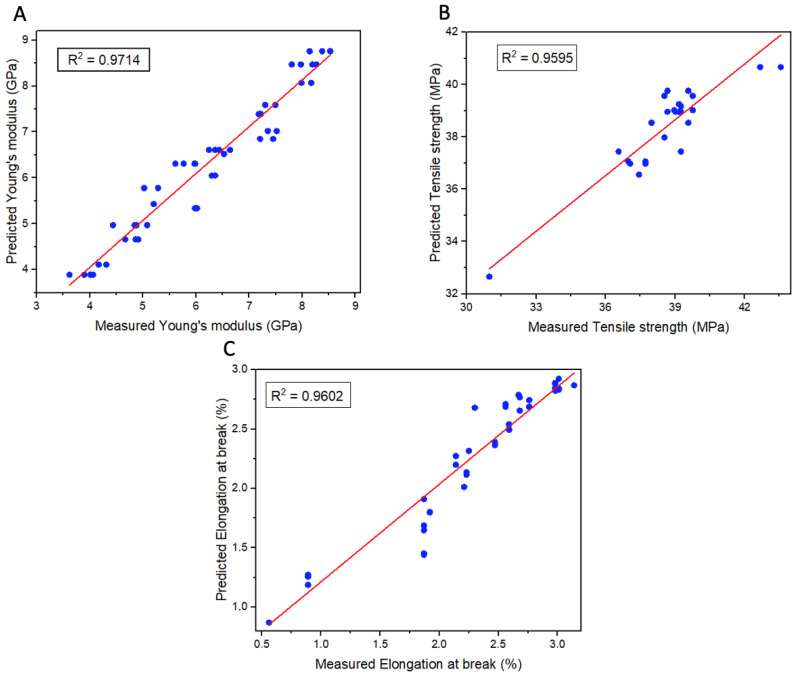
Parity plots showing predicted values using the best regression models for each property vs. the measured values for: (**A**) Young’s modulus; (**B**) tensile strength; (**C**) elongation at break.

Figure 8 shows the parity of the predicted distribution of values versus the measured data for the three mechanical properties investigated. Each property is shown using the regression model with the best specific coefficient of determination (*R*^2^). For Young’s modulus, an artificial neural network (ANN) model was used, for tensile strength, a decision tree (DT) model was used, and for elongation at break, a support vector machine (SVM) model was used. Reproducibility was measured using 10 simulations of the models under the same conditions selected as optimal. The plots showed a satisfactory correlation between the predicted and measured values, wherein the points clustered around the diagonal line, indicating the optimal fit of the model. Thus, a slope very close to 1.0 was obtained in all cases. The best prediction was found for Young’s modulus, with an *R*^2^ value higher than 0.97. 

In the evaluation of predictive models, it is crucial to select those that can accurately calculate the values of the target variable. Therefore, the minimum square error (*MSE*) and mean absolute error (*MAE*) of the three models were also compared, and the results are shown in Table 8. *MSE* is regarded as one of the most important validation criteria of ML models. The model with the smallest *MSE* value was considered to be the most suitable for property prediction. 

Regarding the Young’s modulus (Table 8), the *MSE* values were lower than 0.3 for all models, with 0.0598 for ANN and 0.0503 for SVM. The errors found herein were smaller than those reported previously for other graphene-based nanocomposites [57,62] (*MSE* ≤ 0.6 [62] and 0.45 [57]). In addition, the error percentage found herein (*MSE* in the range of 0.64–1.0%) was smaller. The *MAE* values were also lower than 0.5, with 0.1876 for ANN and 0.2015 for SVM. These errors were lower than those previously reported in other studies that predicted the mechanical properties of nanocomposites, in which the *MAE* values were around 3 [68] or even higher at about 4.2 [69]. The *MAE* percentages for the different models obtained herein were in the range of 3.54–3.65%. These results corroborated the high potential of the models for accurately predicting the stiffness of the nanocomposites. Focusing on tensile strength (Table 8), the values of *MSE* and *MAE* were significantly higher, up to 3, 2, and 0.7 for the ANN, SVM, and DT models, respectively. In particular, for the best model (DT), the *MSE* and *MAE* percentages for this property were in the range of 0.02–9.11% and 0.2–5.42%, respectively. Thus, the models were less effective in predicting the strength of the nanocomposites, likely because this property did not follow a trend with nanofiller loading, as shown in Figure 2B. Further, the stiffness of the nanocomposites was mainly conditioned by the modulus of each constituent, while the strength depended more on other factors, such as the state of dispersion, orientation of the nanofillers, and interfacial adhesion between the components; therefore, it was more difficult to predicted the tensile strength of the nanocomposites from the strengths of the individual components and their weight ratios. With regard to elongation at break, the trend was similar to that observed for the Young’s modulus, with even lower *MSE* and *MAE* values (in the range of 0.14–0.28% and 2.34–3.02%, respectively).

Overall, it was found that the ANN model was very suitable for predicting the mechanical properties of the hybrid polymeric nanocomposites, and that the SVM model had comparable performance to the ANN model in terms of accuracy and computational efficiency, in agreement with the results reported earlier [57]. This was attributed to the fact that the SVM model required less feature data to generalize the norm and did not include iteration processes as in the ANN model for optimizing weight and bias parameters. 

Table 9 presents the experimental and predicted values of the mechanical properties for the three predictive models (ANN, SVM, and DT). In general, good correspondence between the measured and predicted values was observed, although in a few cases, significant discrepancies were detected. These deviations were more evident in the values predicted for tensile strength, as expected, taking into account the higher *MSE* and *MAE* values obtained. The first value predicted for tensile strength using the ANN model overestimated the experimental value, while the first value predicted using the SVM model was underestimated. The DT model yielded a value closer to the experimental value. The models were less accurate in predicting tensile strength, as it is a more complex property that depends on multiple factors, making it difficult for the models to capture these complex interactions. Young’s modulus and elongation at break are properties strongly related to the microstructural characteristics of the material, hence the ANN, SVM, and DT models were able to handle the non-linear and complex relationships between the microstructural characteristics of the material and its mechanical properties.

## 5. Conclusions

The development of hybrid polymeric nanocomposites is very costly and time consuming. Their behaviors are strongly influenced by their composition, which makes the accurate estimation of their properties a major challenge. In this study, ternary hybrid nanocomposites based on poly(hydroxybutyrate-co-hydroxyvalerate) (PHBV) biopolymer reinforced with GO and montmorillonite nanoclay were prepared via a simple green electrochemical approach followed by solution casting using glacial acetic acid as an alternative to conventional organic solvents. Young’s modulus, tensile strength, elongation at break, and impact toughness were measured by stress–strain and impact tests. The traditional rule of mixtures and the Krenchel equation were applied to predict the experimental data, but the predictions were very poor, with differences up to 95%. In addition, the effectiveness of different machine learning (ML) regression models to predict these mechanical properties was evaluated. ANN, DT, and SVM models were compared and the influence of different factors on the accuracy of the models was analyzed. The ANN model with 800 neurons in a single hidden layer was found to be a reliable and effective model for the prediction of the Young’s modulus, with *R*^2^ values for the training and testing sets of 0.9984 and 0.996, respectively. Furthermore, the *MSE* and *MAE* values of the testing set were minimized to 0.0598 and 0.1876, respectively. Regarding the tensile strength, the DT model stood out vs. the ANN and SVM models, with *R*^2^ values of the training and testing sets equal to 1 and 0.9386, respectively, along with *MSE* and *MAE* values of the testing set of 0.6364 and 0.5140, respectively. For the elongation at break, the SVM model outperformed the ANN and DT models. Upon adjusting C, gamma, and epsilon parameters, *R*^2^ values of 0.9526 and 0.9127 for the training and testing sets were obtained, respectively, along with values of 0.0121 and 0.1011 for *MSE* and *MAE*, respectively. The high correlation coefficient values and small errors obtained compared to former studies on polymer-based multiscale nanocomposites supported the accuracy of the models. Moreover, their good generalization capability was indicated by almost uniform values of the correlation coefficient and mean square error for the training and testing datasets. 

In order to confirm the generalizability of the conclusions drawn, identify possible uncertainties, and assess the applicability of the ML models developed herein in a wider range of situations, new experimental databases, including data from our own research and other research work, with different characteristics in terms of composition and properties are currently being tested and the results will be reported in future articles. Preliminary results indicate that these ML models are also suitable for accurately predicting the properties of other polymer-based multiscale nanocomposites. By conducting further experiments and collecting additional data, we seek to better understand the possible uncertainties and limitations of the models. This will allow us to provide a more comprehensive assessment of the reliability and applicability of machine learning models in different scenarios. This exploration of different systems will enable a better understanding of the capabilities and generalizability of these models in broader situations, thus providing external validation of their performance. Overall, the developed models were found to be suitable for predicting the mechanical properties of the hybrid polymeric nanocomposites, opening new possibilities for future applications and developments, particularly in the biomedical field.

## Figures and Tables

**Table 1:** Mechanical properties of PHVB/GO/nanoclay nanocomposites for different nanofiller concentrations.

Sample	GO (wt%)	Nanoclay (wt%)	Young’s Modulus (GPa)	Tensile Strength (MPa)	Elongation at Break (%)	Impact Strength (MJ/m^3^)
1	0	0	3.52	37.55	3.12	21.33
2	1	0	3.89	38.67	2.98	21.04
3	2	0	4.97	38.96	2.56	18.41
4	3	0	5.78	39.25	2.47	17.67
5	4	0	6.31	39.17	2.21	15.78
6	5	0	6.19	36.5	1.92	12.79
7	0	1	4.11	37.72	2.67	18.36
8	0	2	5.43	37.06	2.25	15.22
9	0	3	6.52	36.98	1.87	12.61
10	0	4	7.39	32.66	0.89	6.34
11	0	5	7.90	30.98	0.56	4.15
12	1	1	4.66	39.76	3.14	22.71
13	1	2	6.05	39.57	2.76	19.99
14	1	3	6.85	38.54	2.14	15.03
15	1	4	8.07	37.98	1.87	12.98
16	2	1	5.34	39.02	3.01	21.34
17	2	2	7.59	40.67	2.68	19.85
18	2	3	8.76	43.56	2.44	19.37
19	3	1	6.61	37.44	2.3	15.69
20	3	2	8.47	42.67	2.59	20.16
21	4	1	7.02	36.56	2.23	14.88

**Table 2:** Performance of the regression DT for predicting the Young’s modulus for different values of the hyperparameters.

Splitter	Min Samples Split	Min Samples Leaf	Max Leaf Nodes	Max Features	*R*^2^ Train	*R*^2^ Test
best	2	1	None	auto	1	0.8461
random	2	1	None	auto	1	0.8461
best	3	1	None	auto	0.9889	0.8248
best	5	1	None	auto	0.7973	0.8506
best	2	2	None	auto	0.7828	0.4550
best	2	1	5	auto	0.8268	0.6842
best	2	1	8	auto	0.9758	0.7810
best	2	1	10	auto	0.9906	0.8248
best	2	1	20	auto	1	0.8461
best	2	1	30	auto	1	0.8461
best	2	1	40	auto	1	0.8461
best	2	1	50	auto	1	0.8461
best	2	1	60	auto	1	0.8461
best	2	1	80	auto	1	0.8461
best	2	1	100	auto	1	0.8461
best	2	1	None	sqrt	1	0.7957
best	2	1	None	log2	1	0.7874

**Table 3:** Performance of the DT regression model for predicting the tensile strength for different values of the hyperparameters.

Splitter	Min Samples Split	Min Samples Leaf	Max Leaf Nodes	Max Features	*R*^2^ Train	*R*^2^ Test
best	2	1	None	auto	1	0.5728
random	2	1	None	auto	1	0.8540
random	3	1	None	auto	0.9819	0.5322
random	5	1	None	auto	0.9483	0.2249
random	2	2	None	auto	0.6415	0.5079
random	2	1	5	auto	0.8724	0.5655
random	2	1	10	auto	0.9902	0.5274
random	2	1	20	auto	1	0.5728
random	2	1	30	auto	1	0.9144
random	2	1	40	auto	1	0.9159
random	2	1	50	auto	1	0.9172
random	2	1	60	auto	1	0.9188
random	2	1	80	auto	1	0.9370
random	2	1	100	auto	1	0.9386
random	2	1	100	sqrt	1	0.9144
random	2	1	100	log2	1	0.9188

**Table 4:** Performance of the SVM model for predicting the tensile strength for different values of the epsilon and gamma hyperparameters.

Epsilon	Gamma	*R*^2^ Train	*R*^2^ Test
0.10	scale	0.9985	0.7678
0.15	scale	0.9966	0.7834
0.20	scale	0.9942	0.8022
0.25	scale	0.9916	0.8157
0.30	scale	0.9887	0.8248
0.35	scale	0.9841	0.8293
0.40	scale	0.9790	0.8294
0.35	0.1	0.9718	0.7786

**Table 5:** Performance of the DT model for different hyperparameters in predicting elongation at break.

Splitter	Min Samples Split	Min Samples Leaf	Max Leaf Nodes	Max Features	*R*^2^ Train	*R*^2^ Test
best	2	1	None	auto	1	0.5531
random	2	1	None	auto	1	0.6511
random	3	1	None	auto	0.9571	0.5939
random	5	1	None	auto	0.8670	0.6025
random	2	2	None	auto	0.7442	0.8786
random	2	1	5	auto	0.8426	0.5838
random	2	1	10	auto	0.9743	0.8863
random	2	1	20	auto	0.9883	0.5346
random	2	1	30	auto	1	0.8787
random	2	1	40	auto	1	0.8769
random	2	1	50	auto	1	0.8102
random	2	1	60	auto	1	0.8894
random	2	1	80	auto	0.9893	0.8770
random	2	1	100	auto	0.9905	0.8878
random	2	1	100	sqrt	1	0.8627
random	2	1	100	log2	1	0.8768

**Table 6:** Performance of the SVM model for different hyperparameters in predicting elongation at break.

Epsilon	Gamma	*R*^2^ Train	*R*^2^ Test
0.01	scale	0.9985	0.6594
0.10	scale	0.9820	0.8057
0.15	scale	0.9686	0.8120
0.20	scale	0.9600	0.8141
0.25	scale	0.9403	0.7940
0.30	scale	0.9103	0.7558
0.35	scale	0.8700	0.6999
0.40	scale	0.8236	0.6250
0.20	0.1	0.9526	0.9127
0.20	0.2	0.9606	0.8228
0.20	0.3	0.9592	0.7745

**Table 7:** *R*^2^ values of the training and testing sets of the three predictive models for the indicated properties.

Property	Coefficient of Determination	ANN	DT	SVM
Young’s modulus	*R*^2^ training	0.9984	1	0.9902
	*R*^2^ testing	0.996	0.8461	0.9728
Tensile strength	*R*^2^ training	0.9941	1	0.9841
	*R*^2^ testing	0.579	0.9386	0.8293
Elongation at break	*R*^2^ training	0.9928	1	0.9526
	*R*^2^ testing	0.8723	0.8909	0.9127

**Table 8:** Comparison of *MSE* and *MAE* of the three predictive models for the indicated mechanical properties.

Property	Metrics	ANN	DT	SVM
Young’s modulus	*MSE*	0.0598	0.3284	0.0503
	*MAE*	0.1876	0.5440	0.2015
Tensile strength	*MSE*	2.9057	0.6364	1.9233
	*MAE*	1.4497	0.5140	1.1139
Elongation at break	*MSE*	0.0346	0.0630	0.0121
	*MAE*	0.1601	0.2080	0.1011

**Table 9:** Comparison of measured and predicted values of the Young’s modulus, tensile strength, and elongation at break for the three models.

Property	ANN		SVM		DT	
	Experimental	Model	Experimental	Model	Experimental	Model
	6.85	7.18	8.47	8.40	8.47	7.59
	4.66	4.49	4.97	4.66	4.97	5.34
Young’s modulus	7.02	7.12	7.02	6.88	7.02	6.61
	5.43	5.33	7.39	7.36	7.39	7.90
(GPa)	8.47	8.77	4.66	4.25	4.66	4.11
	37.44	40.60	42.67	40.25	37.98	38.54
Tensile strength	36.56	37.75	38.96	38.56	30.98	32.66
	39.57	38.22	36.56	36.81	39.57	39.76
	42.67	43.64	32.66	34.36	39.17	39.25
(MPa)	37.98	37.41	39.76	38.97	38.96	39.02
	2.30	2.45	2.14	2.19	2.59	2.68
Elongation at break	2.23	2.14	2.25	2.32	2.56	2.98
	2.76	2.94	1.87	1.69	2.23	2.21
	2.59	2.37	2.47	2.36	0.89	0.56
(%)	1.87	1.41	2.98	2.89	3.14	3.01

## Data Availability

Data will be available on request.

## Supplementary Materials

The following supporting information can be downloaded at: https://www.mdpi.com/article/10.3390/biom13081192/s1, Figure S1: Relationship between the coefficient of determination and the depth of the decision tree for the prediction of the tensile strength; Figure S2. Relationship between the coefficient of determination and the regularization parameter C for the prediction of the tensile strength using SVM regression; Figure S3. Relationship between the coefficient of determination and the depth of the decision tree for the prediction of the strain at break; Figure S4. Relationship between the coefficient of determination and the regularization parameter C for the prediction of the strain at break using SVM regression.
