# Supplementary material for: Synthesis and Characterization of Polyhydroxyalkanoate/Graphene Oxide/Nanoclay Bionanocomposites: Experimental Results and Theoretical Predictions via Machine Learning Models
Source: Biomolecules. 2023 Jul 30;13(8):1192. doi: 10.3390/biom13081192 (PMC10452513; doi:10.3390/biom13081192)
Supplement: Supplementary file 1 [file biomolecules-13-01192-s001.zip › biomolecules-2516073-supplementary.pdf]

Synthesis and Characterization of Polyhydroxyalkanoate/Graphene Oxide/Nanoclay Bionanocomposites: Experimental Results and Theoretical Predictions via Machine Learning Models

Elizabeth Champa-Bujaico [1], Ana M. Díez-Pascual [2,*] and Pilar Garcia-Diaz [1]

[1] Departamento de Teoría de la Señal y Comunicaciones, Universidad de Alcalá, Ctra. Madrid-Barcelona Km. 33.6, 28805 Alcalá de Henares, Madrid, Spain; elizabeth.champa@uah.es (E.C.-B.); pilar.garcia@uah.es (P.G.-D.)
[2] Departamento de Química Analítica, Química Física e Ingeniería Química, Facultad de Ciencias, Universidad de Alcalá, Ctra. Madrid-Barcelona Km. 33.6, 28805 Alcalá de Henares, Madrid, Spain
* Correspondence: am.diez@uah.es

Supporting information

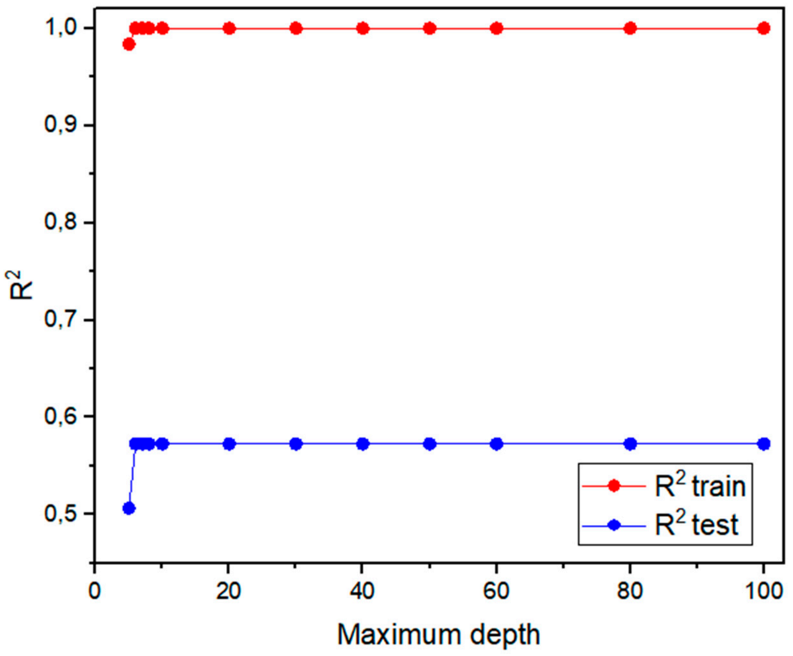

Figure S1. Relationship between the coefficient of determination and the depth of the decision tree for the prediction of the tensile strength

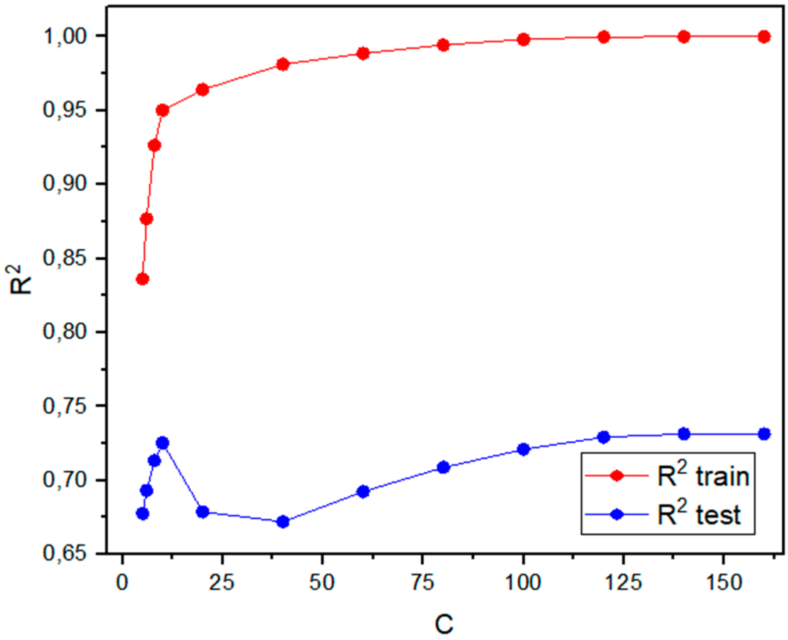

Figure S2. Relationship between the coefficient of determination and the regularization parameter C for the prediction of the tensile strength using SVM regression

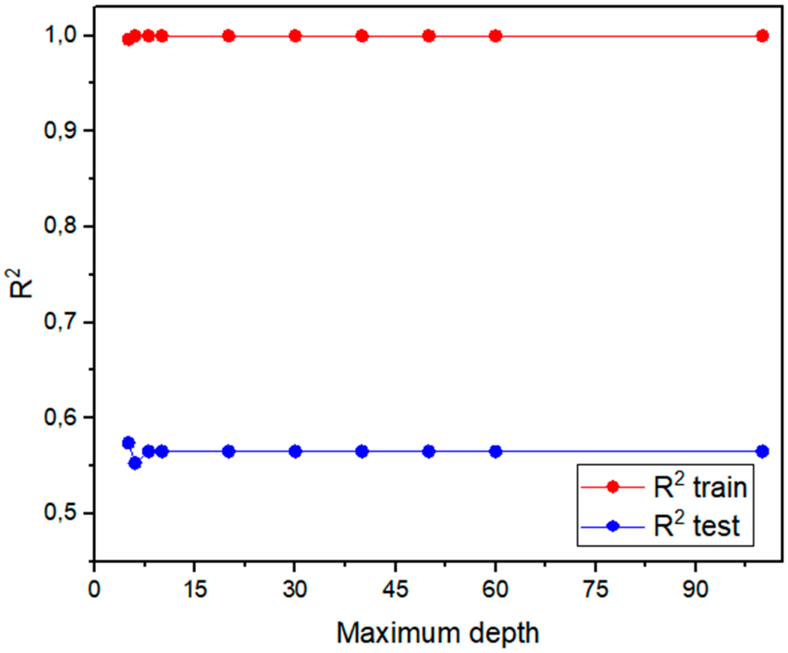

Figure S3. Relationship between the coefficient of determination and the depth of the decision tree for the prediction of the strain at break

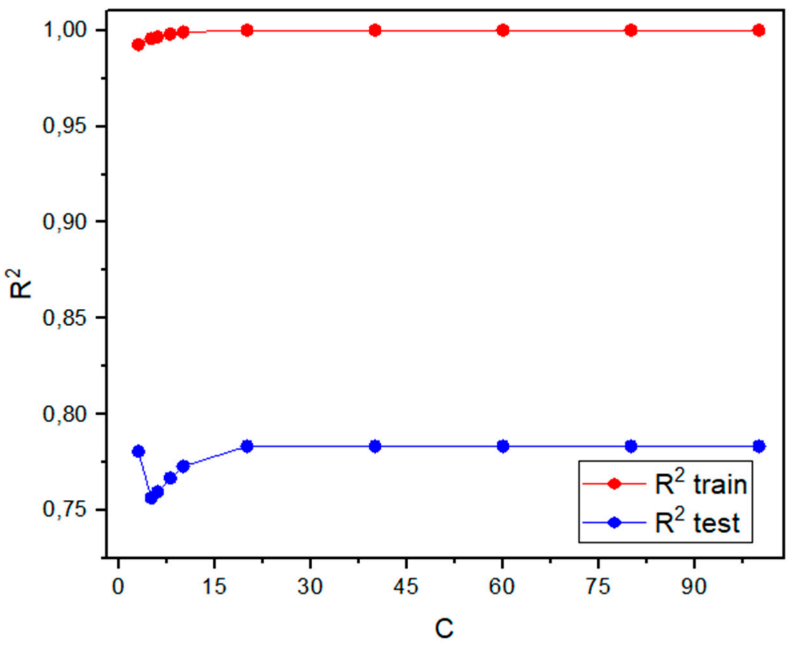

Figure S4. Relationship between the coefficient of determination and the regularization parameter C for the prediction of the strain at break using SVM regression
